# Muscle MR Imaging in Tubular Aggregate Myopathy

**DOI:** 10.1371/journal.pone.0094427

**Published:** 2014-04-10

**Authors:** Valeria Beltrame, Paolo Ortolan, Alessandro Coran, Riccardo Zanato, Matteo Gazzola, Annachiara Frigo, Luca Bello, Elena Pegoraro, Roberto Stramare

**Affiliations:** 1 Department of Medicine, Section of Radiology, University of Padova, Padova, Italy; 2 Department of Cardiac, Thoracic and Vascular Sciences, University of Padova, Padova, Italy; 3 Department of Neurosciences, University of Padova, Padova. Italy; Earl and Christy Powell University, United States of America

## Abstract

**Purpose:**

To evaluate with Magnetic Resonance (MR) the degree of fatty replacement and edematous involvement in skeletal muscles in patients with Tubular Aggregate Myopathy (TAM). To asses the inter-observer agreement in evaluating muscle involvement and the symmetry index of fatty replacement.

**Materials and Methods:**

13 patients were evaluated by MR to ascertain the degree of fatty replacement (T1W sequences) according to Mercuri's scale, and edema score (STIR sequences) according to extent and site.

**Results:**

Fatty replacement mainly affects the posterior superficial compartment of the leg; the anterior compartment is generally spared. Edema was generally poor and almost only in the superficial compartment of the leg. The inter-observer agreement is very good with a Krippendorff's coefficient >0.9. Data show a total symmetry in the muscular replacement (McNemar-Bowker test with p = 1).

**Conclusions:**

MR reveals characteristic muscular involvement, and is a reproducible technique for evaluation of TAM. There may also be a characteristic involvement of the long and short heads of the biceps femoris. It is useful for aimed biopsies, diagnostic hypotheses and evaluation of disease progression.

## Introduction

Tubular Aggregate Myopathy (TAM) is a congenital myopathy characterized by progressive loss of strength, mainly proximal, and frequent cramps and muscle pains induced by exercise. Histologically, it is characterized by tubular aggregates in muscle fibers. According to available data, tubular aggregates are believed to form as a consequence of genetic and/or functional variations, which mainly affect the mechanisms involved in muscle excitation or calcium regulation [1].

To date, no gene responsible for the pathology has been identified: as no specific genetic test is available, clinical and histological examinations, immunohistochemical and histoenzymatic analyses of muscle biopsies, electron microscopy and electromyography are performed for diagnosis.

Evaluation of myopathies/dystrophies by Magnetic Resonance imaging (MR) has always mainly focused on primary and secondary inflammatory skeletal muscle diseases. Several studies report that imaging can also assess the degree of muscle atrophy and the severity of the pathology with high accuracy and sometimes with greater sensitivity than clinical assessment [2]. The different distribution patterns shown by variations in the intensity and severity of muscle involvement may help to achieve differential diagnosis, to identify asymptomatic subjects and to follow up myopathic patients [2]–[7].

This study aimed at evaluating the extent and distribution of fatty replacement and extracellular edema by MR in subjects with TAM, together with assessment of inter-observer agreement, index of symmetry (left and right hemibodies) and a search for the typical signs of the pathologies which MR can provide.

## Materials and Methods

After the review board of the University of Padova had approved this prospective study, our Neurophysiology Center selected 13 patients with TAM (M/F: 8/5, average age 40.4, range 17–67), according to the following criteria:

- clinical condition characterized by progressive loss of strength and/or muscle pains, with cramps and increased hematic CK;
- muscle biopsy revealing tubular aggregates defined as basophilic material in H&E, reddish in color with modified Gomori trichrome stain, intensely reactive to NADH-TR and with negative reaction to SDH; in some cases, diagnosis of TAM was confirmed by electron microscopy;
- absence of other neuromuscular disorders involving loss of strength and tubular aggregates (e.g., limb girdle myasthenia, alcoholic myopathy).

All patients gave their written consent to all tests and to treatment of their data.

### MR assessment

Patients underwent MR assessment (1.5 T, Avanto, Siemens, Erlangen, Germany) with T1-weighted axial scans and Short Tau Inversion Recovery (STIR) sequences (8 mm slice thickness, 150° flip angle) according to the following acquisition parameters: pelvic girdle (FOV: 400, 30 slices, TR: 478 ms, TE: 8.8 msec) and STIR (FOV: 400, 30 slices, TR: 4160 msec, TE: 72 msec) and lower limb (FOV: 400, 32 slices, TR: 596 msec, TE: 8.7 msec) and STIR (FOV: 400, 32 slices,TR: 4160 msec, TE: 72 msec). Images were acquired with a body coil and a simultaneous scan of both sides. 32 slices were acquired in the mid of the thighs and legs, because at this level, the images are more representative of the whole muscle features. Moreover, where the muscle bulk is greater, the muscle involvement could be more clearly analyzed.

Tests generally lasted about 30 minutes.

The skeletal muscles assessed were the following: pelvic girdle (gluteus maximus, medius and minimus, tensor of fascia lata and iliopsoas), anterior compartment of thigh (vastus lateralis, intermedius and medialis, rectus femoris), posterior compartment of thigh (sartorius, gracilis, adductor, semimembranosus, semitendinosus, long and short heads of biceps femoris), anterior compartment of leg (anterior tibial, peroneal, extensor digitorum and extensor hallucis), deep posterior leg compartment (posterior tibial, flexor digitorum and hallucis) and superficial posterior leg compartment (gastrocnemius and soleus).

**Figure 1:**
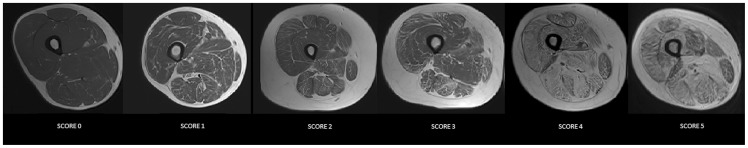
Modified Mercuri's scores, showing progressive grades of muscular fatty replacement.

Areas of signal hyperintensity in T1W sequences were interpreted as areas of fatty infiltration. The extent of fatty replacement and its distribution in muscles were evaluated by staging, specific scores being assigned (modified Mercuri scale) [8] [9] (Fig. 1): normal appearance (score 0); early moth-eaten appearance, with small scattered areas of increased intensity of T1 signal (score 1); late moth-eaten appearance with many discrete areas of increased signal intensity with initial confluence, comprising less than 30% of the volume of the single individual muscle (score 2); late moth-eaten appearance with many quite extended areas of increased intensity of T1, comprising 30–60% of the volume of each individual muscle (score 3); faded, confused appearance, produced by confluent areas of increased signal intensity (score 4); last-stage appearance, with muscle replaced with adipose and connective tissue with increased signal intensity, only a thin muscle fascia preserved, and neuromuscular structures still distinguishable (score 5).

Short Tau Inversion Recovery sequences (STIR) can identify injury in muscles which appear normal in T1 and can clarify the nature of the signal abnormalities common to several muscular disorders caused by various genetic or protein defects[10]. Hyperintense signals in STIR sequences were used to assess the degree of edema, divided into 3 levels (absent, mild, moderate). The distribution of edema was also classified as interfascicular (fluid in interfascicular space) or intrafascicular (fluid in space between individual myofibers). Edema was defined as segmented if it affected less than half of the acquired muscle sections, and comprehensive if it affected more than half of the axial acquisitions. A specific edema score was also used: absent (score 0); mild, intrafascicular (1); mild, intrafascicular, segmented (2); mild, intrafascicular, global (3), moderate intrafascicular, segmented (4); moderate intrafascicular, global (5).

The images of the 13 patients were analysed in a blind study by three doctors with at least two years of experience in musculoskeletal MRI assessment. Each observer evaluated the 26 muscles of patients' lower limbs (total 1014 ratings). Each of the investigated muscles was analyzed separately and, when a different score was assigned to the proximal and distal images, the average score was used.

### Statistical evaluation

In view of the ordinal nature of our data, Krippendorff's coefficient (“alpha”) [11] was used to quantify inter-observer agreement, and 95% confidence limits were calculated by bootstrap sampling [12], in order to take the small sample size into account. The closer the alpha coefficient to 1, the greater the agreement (alpha 0.00–0.20: minimum agreement; 0.21–0.40: intermediate; 0.41–0.60: moderate; 0.61–0.80; substantial: 0.81–1.00, almost perfect) [11].

For symmetry evaluation of left and right (hemibodies), the McNemar-Bowker statistical test [13] was employed (test for dependent samples).

## Results

13 patients were examined for TAM (Tab. 1). Their average age at the onset of symptoms was 17.1 years (minimum 3 years, maximum 66) with an average disease duration of 22.7 years. Most of the patients (9 out of 13) showed autosomal dominant inheritance; the remaining 4 were sporadic cases. Symptoms at onset were variable.

**Table 1:** Clinical features of patients with Tubular Aggregate Myopathy.

Patient	Age/gender	Age at onset of symptoms (yrs)	Duration of illness (yrs)	Presenting symptom	Transmission	CK (U/L)
1.	20/F	6	14	weakness lower limbs	AD	4500
2.	38/F	7	31	weakness lower limbs	AD	1200
3.	46/F	6	40	difficulties in running	AD	2500
4.	43/M	6	37	weakness lower limbs	AD	N/A
5.	34/M	21	13	increased CPK	AD	2000
6.	64/F	6	58	difficulties in running	AD	N/A
7.	17/F	7	10	weakness lower limbs	AD	N/A
8.	41/M	7	34	weakness lower limbs	AD	N/A
9.	28/M	3	25	difficulties in running	S	240
10.	28/M	3	25	difficulties in running	S	130
11.	56/M	46	10	exercise intolerance	AD	1000
12.	43/M	38	5	cramps	S	800
13.	67/M	66	1	weakness of extensor muscles of neck	S	Normal

**Figure 2:**
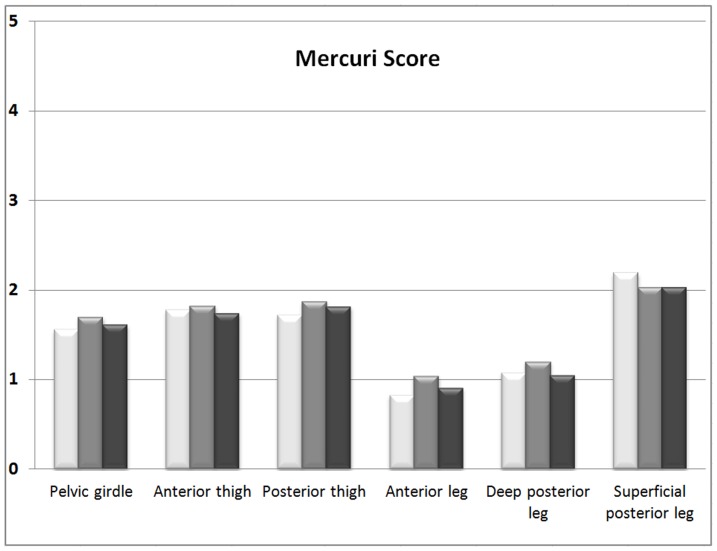
Mercuri scores of muscles divided into areas (three observers).

Analysis of the degree of fatty muscle replacement, according to anatomical area, showed that the superficial posterior leg compartment was the most frequently involved, with an average Mercuri score of 2–2.2. The anterior leg compartment was the least affected (Fig. 2).

**Figure 3:**
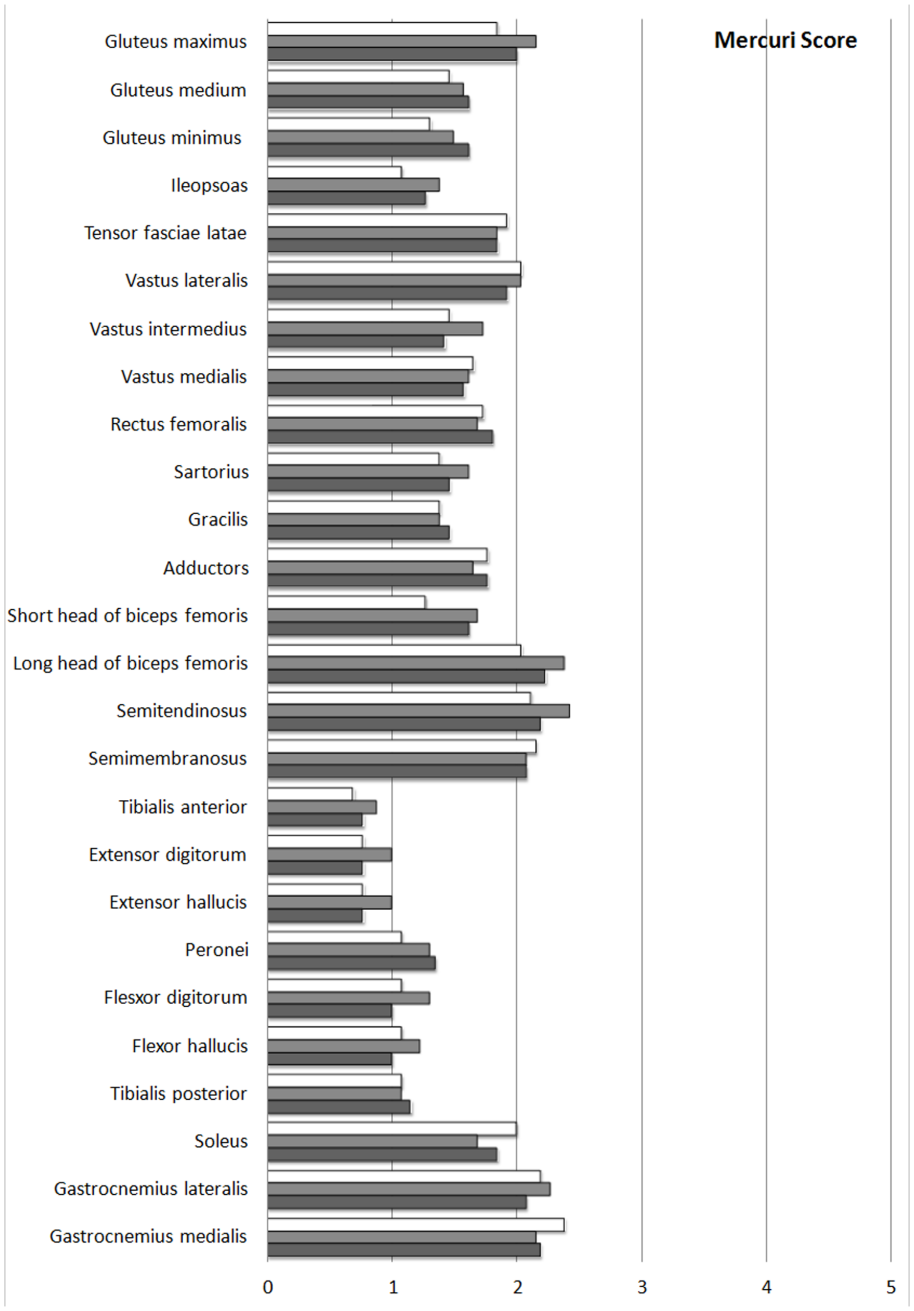
Mercuri scores of individual muscles (three observers).

Analysis of each muscle (Fig. 3) showed that those most affected by fatty involution were the medial gastrocnemius, semimembranosus, gastrocnemius lateralis, semitendinosus, long head of the biceps femoris, vastus lateralis and soleus. Less affected muscles were the iliopsoas, peroneal, flexor digitorum longus and hallucis, posterior tibial, extensors of fingers and hallux, and tibialis anterior (Tab. 2).

**Table 2:** Inter-observer agreement: values of Krippendorf's alpha coefficient of individual muscles with 95% confidence limits.

Muscle	Krippendorff's alpha	LL95CI	UL95CI
Gluteus maximus	0.7517	0.4951	0.9404
Gluteus medius	0.8165	0.6199	0.9616
Gluteus minimus	0.7543	0.5588	0.9152
Iliopsoas	0.8866	0.7968	0.9616
Tensor of fascia lata	0.9870	0.9767	0.9957
Vastus lateralis	0.9649	0.9453	0.9828
Vastus intermedius	0.8975	0.8001	0.9729
Vastus medialis	0.9047	0.8433	0.9562
Rectus femoris	0.9726	0.9531	0.9894
Sartorius	0.9031	0.8486	0.9506
Gracilis	0.9018	0.8382	0.9566
Adductors	0.9060	0.8670	0.9428
Biceps femoris, short head	0.7484	0.6058	0.8779
Biceps femoris, long head	0.9124	0.8399	0.9716
Semitendinosus	0.9167	0.8409	0.9773
Semimembranosus	0.9883	0.9762	0.9979
Anterior tibial	0.8589	0.6409	0.9990
Extensor digitorum	0.9260	0.8409	0.9893
Extensor hallucis	0.9260	0.8424	0.9898
Peroneus	0.9040	0.8112	0.9752
Flexor digitorum brevis	0.3391	−0.0324	0.6544
Flexor hallucis longus	0.3475	−0.0192	0.6659
Posterior tibial	0.9409	0.8815	0.9855
Soleus	0.9000	0.8602	0.9392
Gastrocnemius lateralis	0.9573	0.9192	0.9855
Medial gastrocnemius	0.9667	0.9483	0.9846

As regards the degree of edema, the most afflicted muscle area was the superficial posterior leg compartment, with an average edema score of 0.9.

Most muscles appeared to be only slightly affected by edema; in others, it appeared to be completely absent.

Inter-observer agreement was assessed according to each observer's scores with respect to the parameter “fatty replacement”, as the extent of edema was too small to reach statistical significance. Inter-observer agreement was calculated according to Krippendorff's coefficient. Agreement in all areas was “almost perfect”, the coefficient always being >0.9.

As regards individual muscle assessment, most muscles (21/26) showed “nearly perfect” concordance; 3/26 had “substantial” agreement. Inter-observer agreement was “fair” (alpha <0.35) (Tab. 2) only for the flexor hallucis longus and flexor digitorum longus.

The left and right (hemibodies) overlapped almost perfectly, as confirmed by the McNemar-Bowker test (p = 1).

**Figure 4:**
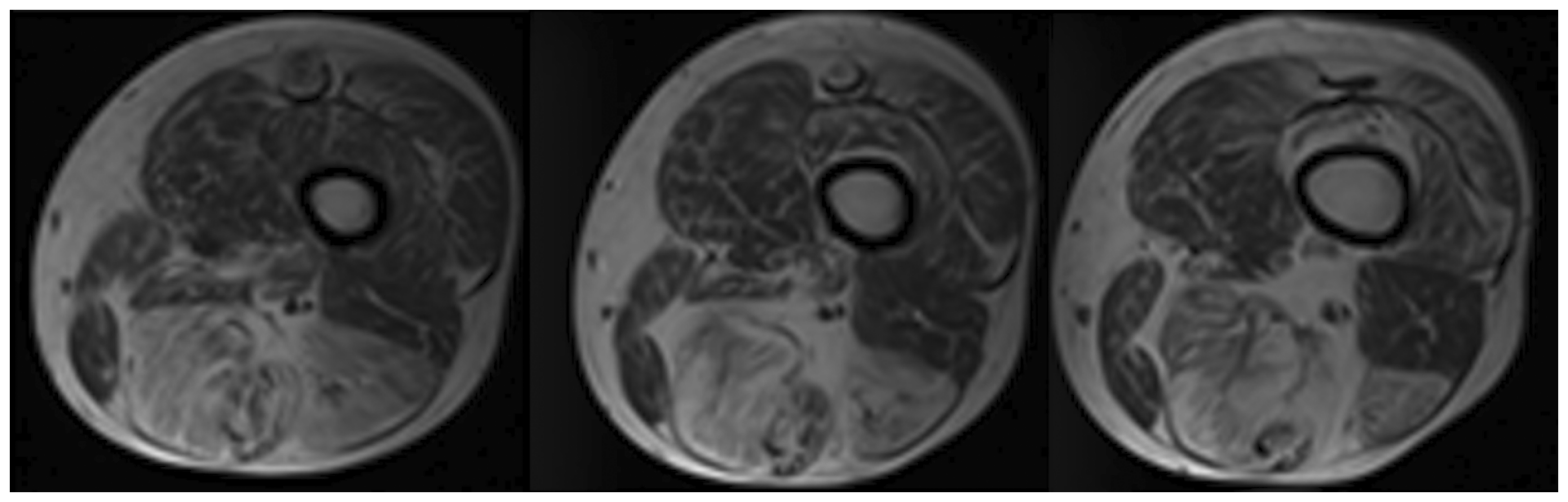
T1W sequence of thigh of same patient in cranio-caudal progression. Different fatty replacement between long head (black arrow) and short head (white arrow) of biceps femoris. Hyperintense appearance, due to fat replacement of long head of biceps femoris, is similar to that of semimembranosus and semitendinosus muscles.

Four out of ten patients showed a different degree of fatty replacement in the short and long heads of the biceps femoris. In particular, the long rather than the short head was more involuted (Fig. 4), as confirmed by Fisher's exact test (p = 0.0004).

**Figure 5:**
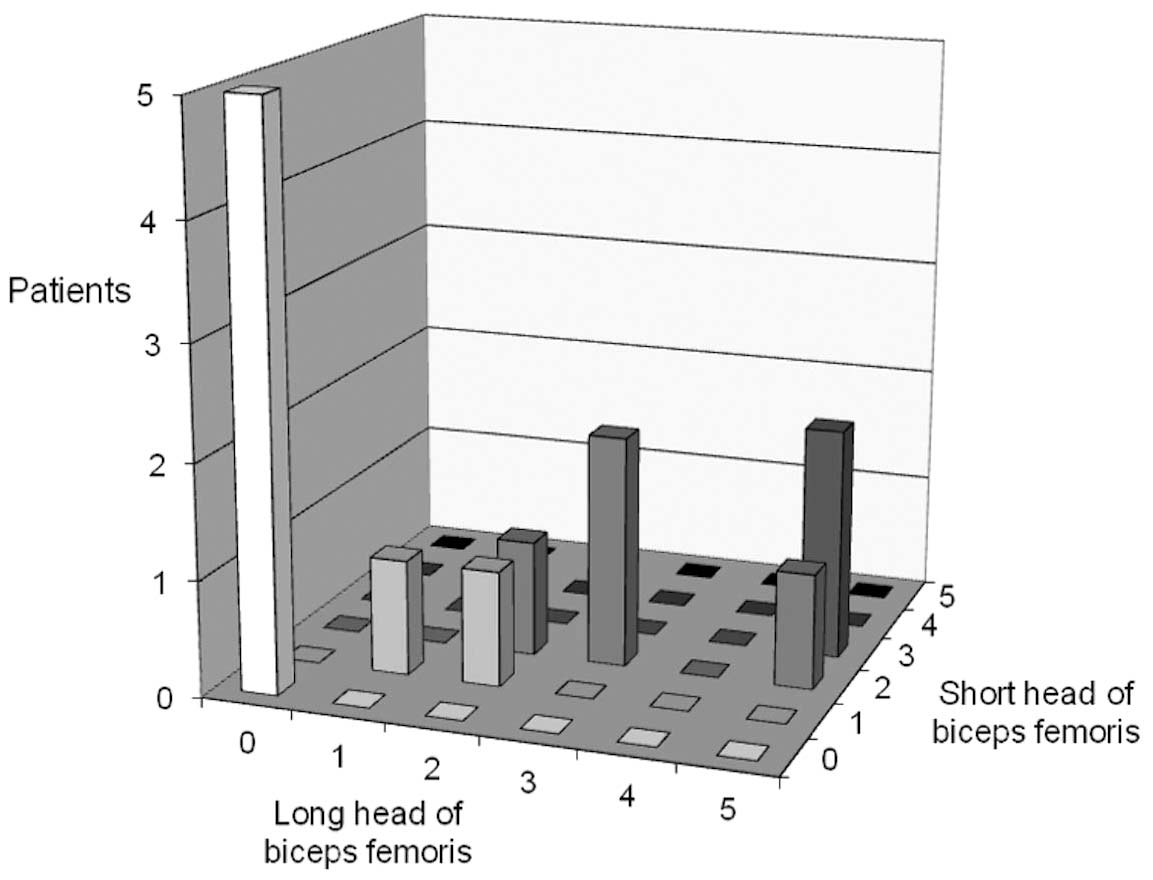
Comparison between degree of fatty replacement (modified Mercuri scale) of long and short heads of biceps femoris in 13 patients. A degree of fatty replacement of long head corresponds to a degree of replacement equal to or less than that of short head. Note in particular how difference between two heads increases with increasing degree of replacement of long head.

The difference in involution between the long and short heads of the biceps femoris increased with greater fatty replacement. In patients with a higher average replacement rate of the lower limb, the long head appeared proportionally much more affected than the short head: this difference increased with the greater severity of the illness (Fig. 5).

## Discussion

Tubular aggregates were described for the first time by Engel in 1966 in the muscle fibers of patients suffering from periodic paralysis and congenital myotonia [10]. These aggregates are predominantly subsarcolemmal inclusions of densly packed membranous tubules, originating from the sarcoplasmic reticulum [1] and associated with various pathologies, both congenital and acquired [11]–[15], such as hypo- or hypercalcemia, periodic paralysis, congenital myotonia, malignant hyperthermia, inflammatory myopathy, alcoholic myopathy and Whipple's disease [16]. It has been proved that tubular aggregates affect calcium homeostasis at subsarcolemmal level and that the sarcoplasmic reticulum is involved in their formation [1]. In the case of TAM, they represent the main histological feature.

Imaging in myopathic patients is useful in clarifying both evolutionary and presymptomatic aspects [2]. MR applications in the study of muscular dystrophies were first employed after experience with CT, which had already made a significant contribution. Cupler's study (1998) of Miyoshi myopathy highlighted the presence of extracellular edema in STIR in the posterior leg compartment at the onset of the disease [17].

Thanks to high definition of anatomical details, T1W sequences can visualize and quantify the extent of fatty muscle replacement which characterizes muscle pathologies and which worsens progressively. Signal hyperintensity in T1W sequences is an expression of muscle fatty replacement. STIR sequences can detect edema in tissues (such as signal hyperintensity), a sign of inflammatory reaction.

In our 13 TAM patients, 41% of the muscles examined did not show any pathological involvement; 17% of them were classified according to a Mercuri score of 1, 14% with a score of 2, 13% with 3, and 8% with 4. Only 7% of the muscles appeared to be completely replaced.

As described in the past for other muscle diseases, the superficial posterior compartment was the worst affected and the anterior compartment the least [18]–[20].

Among individual muscles, the most affected were the gastrocnemius, semimembranosus and semitendinosus, and the tibialis anterior, extensor hallucis longus and extensor digitorum longus the least.

This may indicate that, in myopathies, the process of fatty involution and myofiber degeneration affects the most commonly used muscles, as myocells show greater inability to respond to stress.

Unlike fatty replacement, muscle edema was hardly ever found in the areas analysed. The most affected area was the superficial posterior leg compartment. Other muscle compartments were virtually free of edema. In the case of myositis and also of many dystrophies, the MR aspect differs.

As regards assessment of inter-observer agreement, our data showed good agreement in fatty replacement scores according to the modified Mercuri scale. Only the flexor digitorum longus and flexor hallucis longus in the deep posterior leg compartment showed poor agreement. This was very probably due partly to the difficulty of identifying and isolating these muscles in anatomical MR images, especially in patients who do not display particularly trophic activity, and partly to the small number of patients and low scores found by the three observers.

The literature contains few works identifying MR characteristics typical of some muscle pathologies. Works on type 1 myotonic dystrophy, hyaline body myopathy and girdle dystrophy are rare [18]–[20].

The literature does not contain any records of MR analysis of patients with TAM. In our records, the different replacement level between the long and short heads of the biceps femoris seems peculiar:

Studies have reported that long head of the biceps femoris is more severely affected in Duchenne muscular dystrophy than the short head of the biceps femoris. Akima et al. [21], showed that in Duchenne muscular dystrophy the long head of the biceps femoris was composed of 33% of non-contractile tissue versus 10% in the short head of the biceps femoris.

This pattern has not been described so far in patients with myopathyes. Although limited to 13 patients, this study reports higher progressive fatty replacement of the long head of the biceps femoris compared with the short head, reaching statistical significance. In particular, the short head never reaches a score higher than 3, whereas the long head in three of our patients scored 5. The long head usually has a fatty replacement level similar to that of the semitendinosus muscle, with which it originates at the level of the ischial tuberosity (Fig. 4). The long head, together with the semitendinosus and semimembranosus, makes up the hamstring muscle group which, in addition to extraflexion and rotation of the thigh (like the short head of the biceps femoris), extends the pelvis and aids external rotation of the hip.

As in Duchenne dystrophy, in patients with TAM, the different bio-mechanic of the two biceps femoris heads are probably emphasized.

## Conclusions

MR has several roles to play in the approach to TAM, because it can assess the extent of muscle damage (staging), guide biopsies and assess disease progression.

If confirmed by other studies, the particular finding of more progressive fatty replacement of the long head of the biceps femoris than in the short head may become a feature of TAM muscle involvement.
